# ICTV Virus Taxonomy Profile: *Roniviridae*

**DOI:** 10.1099/jgv.0.001514

**Published:** 2020-10-27

**Authors:** Peter J. Walker, Jeff A. Cowley, Xuan Dong, Jie Huang, Nick Moody, John Ziebuhr, ICTV Report Consortium

**Affiliations:** ^1^​ School of Chemistry and Molecular Biosciences, University of Queensland, St Lucia, Queensland 4072, Australia; ^2^​ CSIRO Agriculture and Food, Queensland Biosciences Precinct, St. Lucia, Queensland 4067, Australia; ^3^​ Yellow Sea Fisheries Research Institute, Chinese Academy of Fishery Sciences, Qingdao, Shandong, PR China; ^4^​ Network of Aquaculture Centres in Asia-Pacific, Bangkok, Thailand; ^5^​ CSIRO Australian Centre for Disease Preparedness, Geelong, Victoria 3220, Australia; ^6^​ Institute of Medical Virology, Justus Liebig University Gießen, Gießen, Hesse 35390, Germany

**Keywords:** *Roniviridae*, *Okavirus*, shrimp, ICTV Report, taxonomy

## Abstract

The family *Roniviridae* includes the genus *Okavirus* for three species of viruses with enveloped, rod-shaped virions. The monopartite, positive-sense ssRNA genome (26–27 kb) contains five canonical long open reading frames (ORFs). ORF1a encodes polyprotein pp1a containing proteinase domains. ORF1b is expressed as a large polyprotein pp1ab by ribosomal frameshifting from ORF1a and encodes replication enzymes. ORF2 encodes the nucleoprotein. ORF3 encodes two envelope glycoproteins. ORFX encodes a putative double membrane-spanning protein. Roniviruses infect shrimp but only yellow head virus is highly pathogenic. This is a summary of the International Committee on Taxonomy of Viruses (ICTV) Report on the family *Roniviridae*, which is available at ictv.global/report/roniviridae.

## Virion

**Fig. 1.**
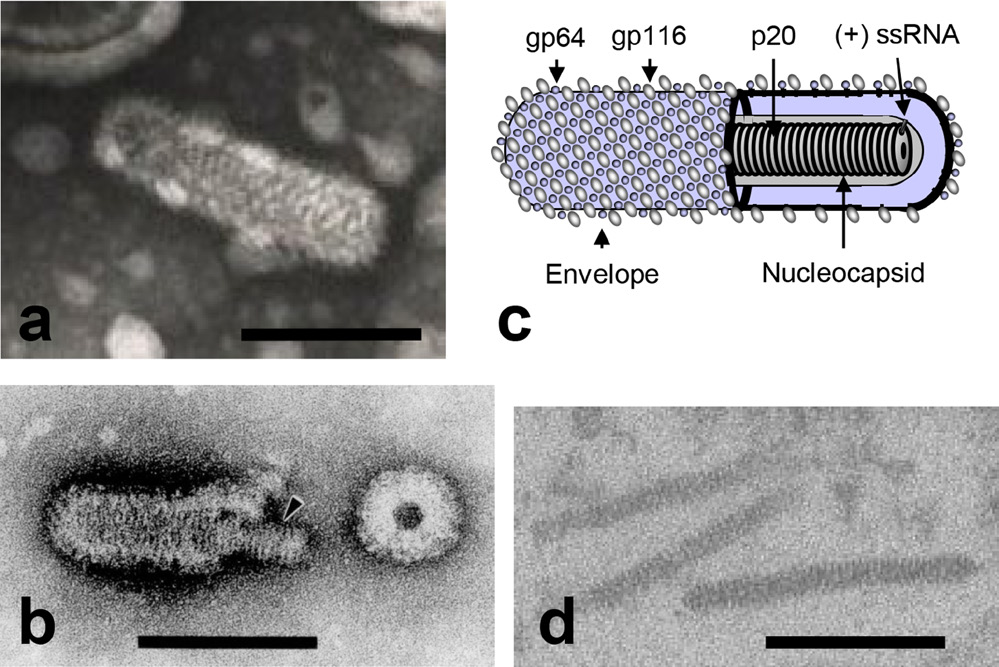
(a) Negative-contrast electron micrograph of gill-associated virus. (b) Negative-contrast electron micrograph of a partially disrupted yellow head virus virion displaying the internal nucleocapsid. (c) Schematic illustration of a ronivirus virion. (d) Thin-section electron micrograph of unenveloped cytoplasmic nucleocapsids in gill-associated virus-infected shrimp cells. The bars represent 100 nm. Courtesy of K. M. Spann, P. Loh, J. A. Cowley and R. J. McCulloch; panels (a), (b) and (c) reproduced with permission from [2].

Virions are enveloped, rod-shaped particles (40–60 nm in diameter and 150–200 nm in length) containing three structural proteins [1] (Table 1, Fig. 1). The nucleoprotein (p20) complexes with the RNA genome to form the helical nucleocapsid. Two transmembrane glycoproteins (gp116 and gp64) form prominent peplomers on the virion surface.

**Table 1.** Characteristics of members of the family *Roniviridae*

Typical member:	gill-associated virus (AF227196), species *Gill-associated virus*, genus *Okavirus*
**Virion**	Enveloped, rod-shaped particles 150–200 nm in length and 40–60 nm in diameter with a helical nucleocapsid composed of the nucleocapsid protein (p20); the lipid envelope contains two transmembrane glycoproteins (gp64 and gp116)
**Genome**	Positive-sense, single-stranded RNA (26–27 kb) containing 5 or 6 long open reading frames
**Replication**	Cytoplasmic; nucleocapsids bud at membranes of the endoplasmic reticulum/Golgi complex to form mature virions
**Translation**	From a nested set of 5′-capped and 3′-co-terminal polyadenylated mRNAs transcribed from genomic RNA
**Host Range**	Penaeid shrimp are natural hosts; experimental infection reported in penaeid and palaemonid shrimp of various species
**Taxonomy**	Realm *Riboviria*, kingdom *Orthornavirae*, phylum *Pisuviricota*, class *Pisoniviricetes,* order *Nidovirales*; the subfamily *Okanivirinae* includes the genus *Okavirus*, the subgenus *Tipravirus* and three species

## Genome

**Fig. 2.**
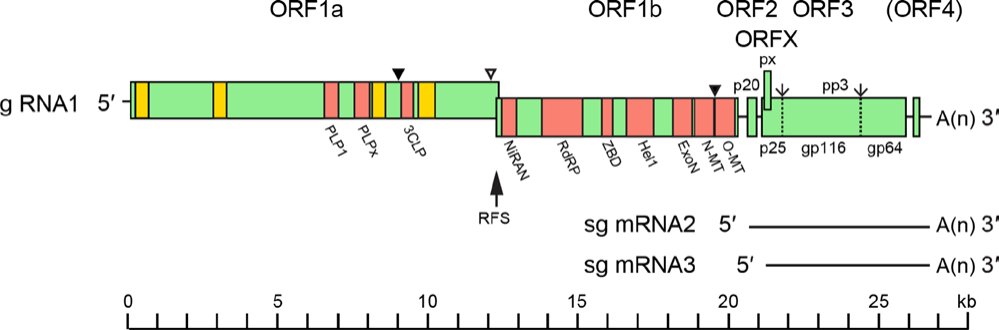
Yellow head virus genome (g RNA1, 26 662 nt) and the two 3′-coterminal sub-genomic RNAs, sg mRNA2 and sg mRNA3. ORF1a hydrophobic regions HD1–HD4 (yellow). Functional domains (pink) - ORF1a: 3C-like protease (3CLP) and papain-like protease domains PLP1 and PLPx; ORF1b: nidovirus RdRP-associated nucleotidyltransferase (NiRAN); RNA-directed RNA polymerase (RdRP); cysteine- and histidine-rich zinc-binding domain (ZBD); superfamily 1 helicase (Hel1); exoribonuclease (ExoN); guanosine N7-methyltransferase (N-MT); and ribose 2′-O-methyl transferase (O-MT). ORF2 encodes the nucleoprotein (p20). ORF3 encodes a precursor polyprotein (pp3), which undergoes post-translation processing to generate envelope glycoproteins (gp116 and gp64) and an N-terminal triple membrane-spanning fragment (p25). ORFX is an alternative reading frame in ORF3 encoding a small double membrane-spanning protein (px). RFS - ribosomal frameshift site upstream of the ORF1a stop codon that allows translation of pp1ab. Known (▼) and likely (∇) sites of proteolytic cleavage of pp1a and pp1ab; signal peptidase type 1 cleavage sites in pp3 (↓).

The ronivirus genome is a linear, positive-sense ssRNA (26–27 kb) with a 5′-methylated cap and 3′-polyadenylated tail (Fig. 2) [2]. The genome contains five canonical long open reading frames (ORFs), which, in order from the 5′-terminus, include: ORF1a, and the overlapping ORF1b, encoding replicase enzymes; ORF2 encoding the nucleoprotein (p20); ORF3 encoding the precursor polyprotein (pp3) from which the envelope glycoproteins gp116 and gp64 are derived; and alternative ORFX, which commences three nucleotides downstream of the pp3 initiation codon and encodes a putative small double membrane-spanning protein (px). In gill-associated virus, ORF3 is followed by ORF4 but it is severely truncated in other roniviruses and evidence for its expression is poor.

## Replication

Ronivirus replication is cytoplasmic. Elongated nucleocapsids are visible in infected cells and bud at membranes of the endoplasmic reticulum/Golgi complex to form mature virions. During infection, genome length RNA (g RNA1) and two 3′-coterminal subgenomic mRNAs (sg mRNA2 and sg mRNA3) are transcribed, each with a 5′-methylated cap and a 3′-poly(A) tail [3]. Double-stranded RNAs of equivalent size appear to be replicative intermediates. Ronivirus g RNA1, sg mRNA2 and sg mRNA3 each initiate with a 5′-AC dinucleotide and lack a common leader sequence. Roniviruses appear to differ from coronaviruses and arteriviruses by not using a discontinuous transcription, but rather a continuous transcription strategy similar to that utilized by toroviruses.

## Taxonomy

Current taxonomy: ictv.global/taxonomy. The family *Roniviridae* includes the genus *Okaviru*s with the species *Yellow head virus*, *Gill-associated virus* and *Okavirus 1*. Viruses in these species represent only three of eight okavirus genotypes that have been identified in penaeid shrimp [4, 5]. Yellow head virus (species *Yellow head virus*) is assigned to genotype 1 with two subtypes (1a and 1b), gill-associated virus (species *Gill-associated virus*) is assigned to genotype 2 and yellow head virus-8 (species *Okavirus 1*) is assigned to genotype 8. Roniviruses are most closely related to other nidoviruses infecting arthropods, including members of the families *Mesoniviridae* (from mosquitoes) and *Euroniviridae* (from crustaceans).

## Resources

Current ICTV Report on the family *Roniviridae*: ictv.global/report/roniviridae
